# Validity and Reliability of a New Wearable Chest Strap to Estimate Respiratory Frequency in Elite Soccer Athletes

**DOI:** 10.3390/sports12100277

**Published:** 2024-10-12

**Authors:** Adriano Di Paco, Diego A. Bonilla, Rocco Perrotta, Raffaele Canonico, Erika Cione, Roberto Cannataro

**Affiliations:** 1 Lung Unit Division, San Rossore Clinic, 56122 Pisa, Italy; dipaco@sanrossoresportvillage.it; 2 Research Division, Dynamical Business & Science Society—DBSS International SAS, Bogotá 110311, Colombia; dabonilla@dbss.pro; 3 Hologenomiks Research Group, Department of Genetics, Physical Anthropology and Animal Physiology, University of the Basque Country (UPV/EHU), 48940 Leioa, Spain; 4 Universidad Católica San Antonio de Murcia Campus de Murcia, Guadalupe, 30107 Murcia, Spain; rperrotta@alu.ucam.edu; 5 Empoli Football Club, 50053 Empoli, Italy; 6 Dietetic and Sports Medicine Unit, Luigi Vanvitelli University, 80138 Napoli, Italy; raffaele.canonico@policliniconapoli.it; 7 Department of Pharmacy, Health and Nutritional Sciences, University of Calabria, 87036 Rende, Italy; erika.cione@unical.it; 8 Galascreen Laboratories, University of Calabria, 87036 Rende, Italy

**Keywords:** respiratory physiological phenomena, exercise physiology, work capacity evaluation, exercise intensity, breath frequency

## Abstract

Assessing respiratory frequency (*f*_R_) is practical in monitoring training progress in competitive athletes, especially during exercise. This study aimed to validate a new wearable chest strap (wCS) to estimate *f*_R_ against ergospirometry as a criterion device in soccer players. A total of 26 elite professional soccer players (mean [standard deviation]: 23.6 [4.8] years; 180.6 [5.7] cm; 77.2 [5.4] kg) from three Italian Serie A League teams participated in this cross-sectional study. The sample included attackers, midfielders, and defenders. *f*_R_ was assessed during a maximal cardiopulmonary exercise test (CPET) on a treadmill using (i) a breath-by-breath gas exchange analyzer (Vyntus^®^ CPX, Vyaire Medical) and (ii) a novel wCS with sensors designed to assess breath frequency following chest expansions. Pearson’s correlation coefficient (*r*), adjusted coefficient of determination (aR^2^), Bland–Altman plot analysis, and Lin’s concordance correlation coefficient (ρ_c_) were used for comparative analysis (correlation and concordance) among the methods. The repeated measures correlation coefficient (r_rm_) was used to assess the strength of the linear association between the methods. The intraclass correlation coefficient (ICC) and the Finn coefficient (r_F_) were used for inter-rater reliability. All statistical analyses were performed within the R statistical computing environment, with 95% confidence intervals (95% CIs) reported and statistical significance set at *p* < 0.05. A total of 16529 comparisons were performed after collecting the CPET data. The robust time series analysis with Hodges–Lehmann estimation showed no significant differences between both methods (*p* > 0.05). Correlation among devices was statistically significant and very large (*r* [95% CI]: 0.970 [0.970, 0.971], *p* < 0.01; aR^2^ [95% CI]: 0.942 [0.942, 0.943], *p* < 0.01) with strong evidence supporting consistency of the new wCS (BF_10_ > 100). In addition, a high concordance was found (ρ_c_ [95% CI]: 0.970 [0.969, 0.971], bias correction factor: 0.999). Vyntus^TM^ CPX, as a standard criterion, showed moderate agreement with wCS after Bland–Altman analysis (bias [95% lower to the upper limit of agreement]; % agree: 0.170 [−4.582 to 4.923] breaths·min^−1^; 69.9%). A strong association between measurements (r_rm_ [95% CI]: 0.960 [0.959, 0.961]), a high absolute agreement between methods (ICC [95% CI]: 0.970 [0.970, 0.971]), and high inter-rater reliability (r_F_: 0.947) were found. With an RMSE = 2.42 breaths·min^−1^, the new wCS seems to be an valid and reliable in-field method to evaluate *f*_R_ compared to a breath-by-breath gas exchange analyzer. Notwithstanding, caution is advised if methods are used interchangeably while further external validation occurs.

## 1. Introduction

Over the past three decades, athletic training has undergone substantial transformation, largely driven by advancements in technology. Within the realm of athletic performance evaluation, the identification of a dependable biomarker induced by exercise stress [1] or a variable linked to fatigue [2] holds paramount importance. In this sense, monitoring training load is crucial for understanding and optimizing the physiological and psychological responses to physical exercise [3].

This monitoring helps fine-tune training programs, ensuring an appropriate balance between stress and recovery to promote positive adaptations while minimizing the risk of chronic allostatic overload [4]. Allostatic load indicates how different physiological systems interact, such as the immunological, endocrine, and cardiovascular systems, to comprehend how long-term stress affects health and disease development [5]. This approach allows for a more effective structuring of the training program in all its components (frequency, intensity, loads), while also evaluating external factors such as nutrition, supplements, and the quality and quantity of sleep. In this context, the role of the sports scientist is becoming increasingly important, as a professional who can integrate feedback from devices and experts to optimize performance.

The assessment of the cardiorespiratory response involves the development and validation of instruments and devices for both laboratory and field use. While heart rate serves as a recognized marker of vagal activity [6], heart rate variability (HRV) offers considerable versatility in providing insights into exercise-induced physiological stress, thereby enabling the monitoring of training load [7]. Evaluating the cardiac response, particularly HRV, using wearable devices, including real-time monitoring, has shown its reliability as a dependable field instrument [8,9]. However, it is important to note that these instruments do not directly facilitate on-field evaluation. Similarly, evaluating the ventilatory response adds complexity, particularly because it currently relies on laboratory tests and gas exchange data analyzers, despite its fundamental importance, although some tools for use in the field are starting to become available. The gold standard for performance assessment remains the cardiopulmonary exercise test (CPET), allowing examination of cardiac, ventilatory, and metabolic responses within a controlled lab environment. However, this test is confined to lab settings and not applicable for daily training at a low cost [10] unless indirect tests are used such as the Cooper run test or Shuttle run test [11]. Additionally, the role of respiratory rate might be significant, given its potential for dynamic evaluation. Evidence suggests its correlation with perceived effort and respiratory volumes [12,13,14].

In soccer, previous research has established a link between *f*_R_ and exercise capacity response [15,16]. It is worth noting that a large correlation between *f*_R_ and perceived effort has been reported [12]. In this sense, several methods, such as using a smart facemask [17], recording breathing sounds with a microphone [18], and new algorithms [19], have been developed for respiratory monitoring during exercise. Notwithstanding, there is no doubt that practicing sports while wearing a mask that covers the entire face significantly affects performance, and some other devices are susceptible to bias if signals can be affected by motion.

Therefore, the development of novel respiratory wearable devices represents a promising area of research considering their low invasiveness, good integration in Internet of Things and wearable systems, low energy consumption, and low cost, as stated by Massaroni et al. (2019) [20]. Hence, this study aimed to validate a new wearable chest strap (wCS) to assess respiratory frequency in real-time during an exhaustion test for elite Italian soccer players. This would represent the first step in the development of a device that allows sports scientists to monitor the respiratory frequency, and therefore the training load, in an easy-to-use manner with significantly lower costs while maintaining validity and high reliability when compared to the gold-standard breath-to-breath gas analyzers.

## 2. Materials and Methods

### 2.1. Study Design

A cross-sectional study was conducted on a single-point measurement in elite soccer players following recommendations on the use of exercise testing in clinical practice [21]. The findings are reported following the Strengthening the Reporting of Observational Studies in Epidemiology (STROBE) statement [22].

### 2.2. Setting

The study was conducted between September and October of 2022. The research was performed during the season 2022–2023 of the Serie A. All the measurements were taken in a single session. Evaluations were conducted between 9:00 and 17:00 (CET), always operate in the same place and always use the same device.

### 2.3. Participants

Twenty-six elite professional soccer players from three different soccer teams belonging to the Italian Serie A (23.6 [4.8] years; 180.6 [5.7] cm; 77.2 [5.4] kg; BMI 23.7 [1.0] kg·m^−2^) participated in the study. The sample comprised forwards, midfielders, and defenders who underwent evaluation upon completion of the pre-season schedule of routine medical assessments. As anticipated, none of them reported any smoking habits or significant pathological conditions. At the time of testing, all participants were injury-free for at least three months and had not reported serious musculoskeletal injuries in the last two years. Furthermore, they were briefed on the experimental procedures and provided their consent before taking part. While the participants were unaware of the ultimate objective of this study, they willingly signed an informed consent form allowing their data to be used for research. Ethical approval was granted by the University of Calabria’s scientific and ethics review board (UCALPRG 0076328, 19102022).

### 2.4. Variables

The selected variables in this study included demographics (body mass [BM, kg], stature [cm] and age [years]) and respiratory frequency (*f*_R_, breaths·min^−1^).

### 2.5. Data Sources/Measurement

Prior to the assessment, the athletes performed a three-minute group warm-up consisting of running at 8–10 km per hour, which corresponded to the first three minutes of the test record.

#### 2.5.1. Exercise Test

An incremental symptom-limited exercise test was conducted as reported previously [23]. Following a short warm-up, the participants initiated the test at a speed of 8 km/h, with no slope variation during the test. The protocol involved a “ramp” phase characterized by a gradual speed increment of 1 km/h every 60 s. The test continued until the participant reached failure, which they self-reported. Participants were equipped with a heart rate sensor (Polar H10, Polar, Kempele, Finland).

#### 2.5.2. Novel Wearable Device Description

**Figure 1:**
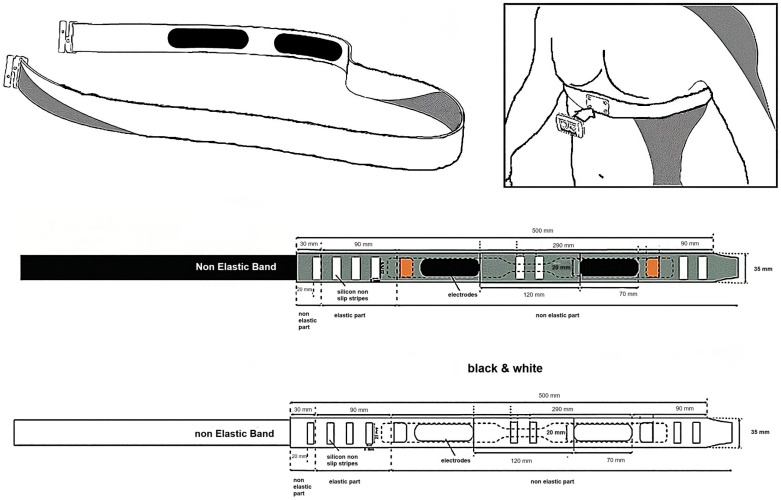
Novel wearable chest strap to measure respiratory frequency.

**Figure 2:**
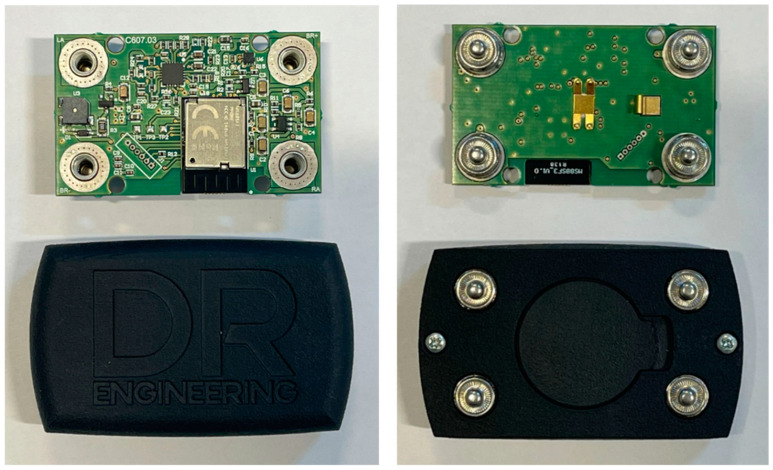
Electronic board, both sides, with and without built-in cover.

The system comprises two components: the chest strap (Figure 1) and the accompanying reading/communication module (Figure 2). The electronic module must be attached to the strap to ensure proper functioning. The strap features two distinct sides: an inner side equipped with electrodes for heart rate measurement, requiring direct contact with the body, and an outer side with four pressure contacts for attaching the transmission module.

(i) Moisten the heart rate electrodes by dampening the sensors located on the inner side of the chest strap.
(ii) Secure the strap around the chest, ensuring the four front contacts are centered. Adjust for comfort, making sure it is neither too tight nor likely to slip.
(iii) Attach the transmitter module to the strap using the clip.

The transmission component consisted of a plastic case (Nylon PA11), which was non-conductive and non-shielding from an electrical standpoint. This case contained an electronic board onto which the Bluetooth Low Energy (BLE) radio transceiver module was placed. Between the upper and lower sections of the case, there was an NBR rubber sealing gasket that prevented the penetration of potential pollutants into the device. The device was mechanically and electrically connected by using four snap contacts that were welded onto tin-plated steel spacers (in compliance with the RoHS and REACH directives). This connection was established with a completely passive fabric band equipped with two electrodes for heart rate monitoring and a resistive strain gauge sensor used for breath detection. When activated, the electronic card cyclically read the analog signals generated by the sensors within the fascia’s structure. After appropriate filtering was applied to eliminate unused signal components, communication of the relevant data occurred with a remote collection station. This communication took place via radio waves utilizing the BLE system operating at 2.4 GHz.

The transmission system employs the Bluetooth Long Range mode, enabling it to cover greater distances compared to the standard BLE protocol. Verified distances in open-air settings are around 150 m or more, while adhering to radiated power levels in compliance with prevailing regulations. The electronic card situated within the module attached to the elastic band is composed of the following distinct sections:

- Transmission module with an integrated BLE radio system;
- Heart rate reading module;
- Respiratory signal amplification section;
- Inertial system for energy-efficient operation control;
- Auxiliary acoustic signaling system.

Heart rate detection relies on a microchip specially designed for this purpose. It does not emit radio frequency signals, being predominantly analog and tailored for applications with very low power consumption. There are no quartz or timed systems that could act as additional sources of radiated signals in the surroundings. Following band-pass filtering, signals captured by the analog front end are processed using the renowned Pan–Tompkins algorithm [24]. This algorithm identifies R peaks related to the PQRST envelope, thereby determining heart rate.

The respiratory rate reading involves an analog operational amplifier. Its primary function is to filter and amplify signals detected by the strain gauge sensor. The output of this amplifier is directly read by the microcontroller integrated with the radio module. After appropriate low-pass filtering to eliminate disturbances caused by belt movement that could distort respiratory readings, a threshold algorithm is utilized for calculating the respiratory rate. Additionally, the filters employ solely passive components such as resistors and capacitors. There are no high-frequency digital signals or systems emitting electromagnetic waves into the environment. To optimize battery life, the board incorporates an energy management strategy that entails completely deactivating the radio frequency section during periods of board inactivity. This function is carried out by an analog inertial system utilizing a MEMS accelerometer. In Table 1, we report more technical information of the device.

#### 2.5.3. Data Acquisition

Participants wore a face breathing mask connected with a fast-responding gas analyzer (Vyntus^®^, Vyaire Medical, Chicago, IL, USA). In addition, they wore the new wearable chest strap (wCS), equipped with sensors to read HR and *f*_R_, during an incremental cardiopulmonary exercise test (CPET) on a treadmill (Runrace 900, Technogym, Gambettola, Italy). The breathing frequency was recorded for the entire test duration, checking inhaled and exhaled gasses breath by breath without mediating any values by the flow meter and fast-responding gas analyzer. Before performing the test, each participant underwent spirometry to obtain a flow–volume loop. Based on the forced expiratory volume in 1 s (FEV1), the maximum voluntary ventilation was determined for the CPET.

### 2.6. Sample Size

Professional soccer players of three Italian Serie A League teams were considered potentially eligible participants of this study. After the call and intention to participate, a convenience non-probabilistic sample of 26 elite players from Empoli FC, Cagliari Calcio, and SSC Napoli were obtained.

### 2.7. Statistical Analysis

Descriptive statistics were expressed as means (standard deviations), as recommended for biomedical research articles [25,26]. Based on previously published recommendations to analyze time series data collected from direct-reading instruments [27], a robust version of a two-sample *t*-test was utilized to adjust for autocorrelation properly [28]. The R package ‘robts: Robust Time Series Analysis’ was used to this end. We evaluated the relationship between measured (Vyntus^®^) and estimated (new wearable chest strap) values of respiratory frequency by using all data measurements during the CPET through a Bayesian correlation analysis [29,30]. This was carried out as all possible values of the correlation were equally likely. Following current recommendations in sport science [31], we report not only Pearson’s correlation coefficient but also the likelihood ratio (also known as Bayes Factor [BF]) and the corresponding 95% credible interval (95% CrI), which is the most widely accepted measure to quantify how much evidence a data set provides for a hypothesis. In our case, the BF was expressed as BF_10_ to grade the intensity of the evidence that the data provided for H_1_ versus H_0_ (where H_0_ is the null hypothesis and H_1_ is the alternative hypothesis that assumes an effect is present). Additionally, the adjusted coefficient of determination (aR^2^) and Lin’s concordance correlation coefficient (ρ_c_) were used for comparative analysis between the new wCS and breath-to-breath ergospirometry values (Vyntus^®^ as a criterion). The mean absolute error (MAE), percentage of absolute error (%error), and root mean squared error (RMSE) were also calculated. The repeated measures correlation coefficient (r_rm_) was used to assess the strength of the linear association between the methods. In contrast, the intraclass correlation coefficient (ICC) with its corresponding 95% confidence interval (95% CI) and the Finn coefficient (r_F_) were used for inter-rater reliability. In addition, Bland–Altman analysis was used for the concordance analysis between the chest strap and ergospirometry assessment. This analysis determines whether the two measurement methods agree sufficiently to be declared interchangeable (D = X − Y). The mean of these differences represents the systematic error (bias), while the variance in these differences (1.96 SD) measures the dispersion of the random error. All statistical analyses were performed within the v4.2.3 R statistical computing environment [32] with a statistical significance of *p* < 0.05.

## 3. Results

**Figure 3:**
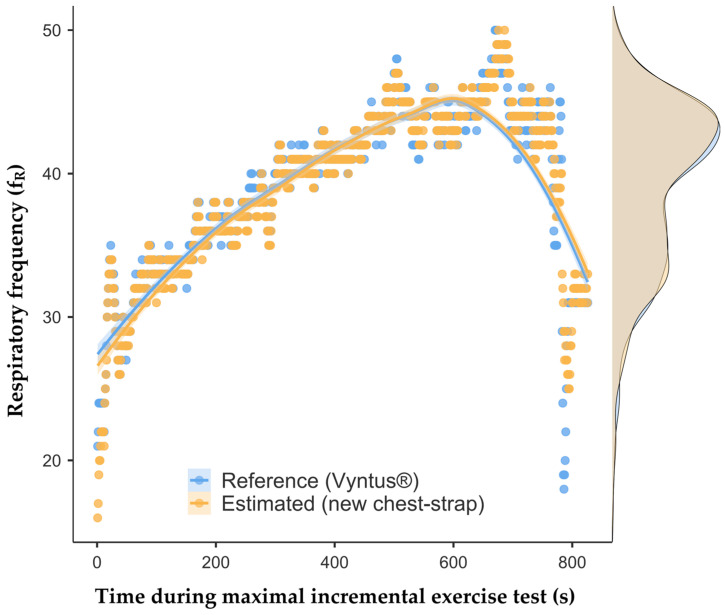
Measured and estimated respiratory frequency (*f*_R_) values. The *f*_R_ is reported in breaths·min^−1^. The scatter plot shows individual measurements over time, with a smooth regression line highlighting the trend for both devices.

All data measurements were obtained from the 26 elite soccer players (23.6 [4.8] years; 180.6 [5.7] cm; 77.2 [5.4] kg) from three Italian Serie A League teams. In total, 16,529 comparisons were performed after collecting data during the maximal CPET. The robust time series analysis with Hodges–Lehmann estimation indicated no significant difference between the estimated and measured time series of *f*_R_ (*p* > 0.05). The distribution of *f*_R_ (illustrated on the right in Figure 3) indicates a close alignment between the reference and estimated values across the entire duration of the test.

**Figure 4:**
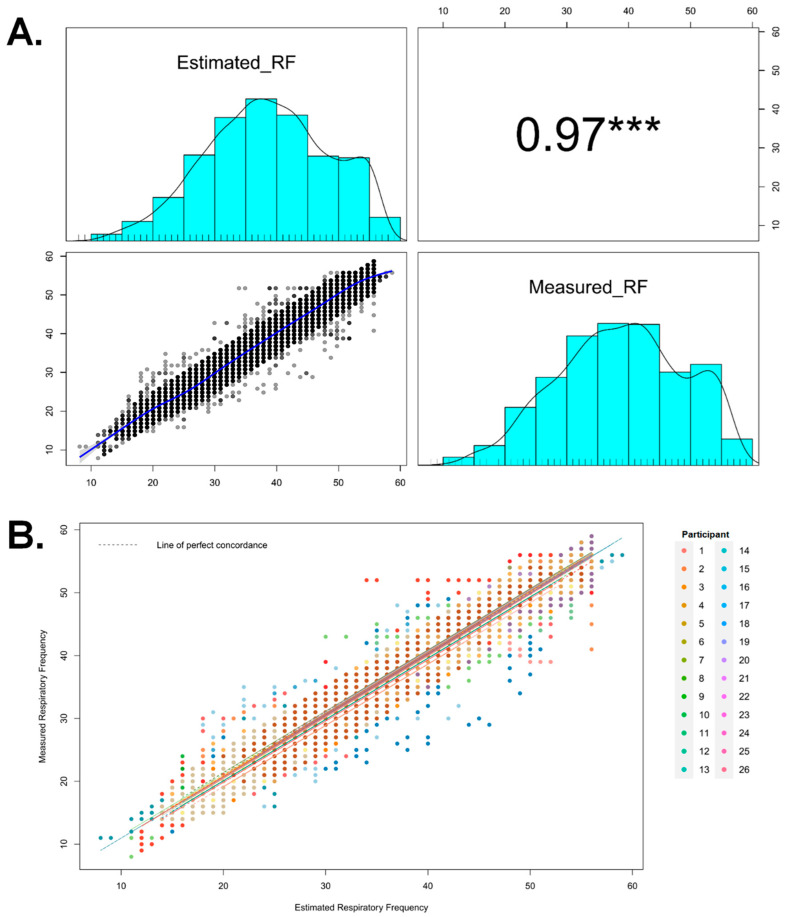
(**A**) Pairwise scatter plot matrix, distribution, and Pearson correlation coefficient. The correlation plot includes histograms, density distributions, and a smooth regression line of the estimated and measured respiratory frequency (*f*_R_) values. *** Statistical significance at *p* ≤ 0.001. (**B**) Repeated measures correlation concordance plot for each participant. Separate parallel lines are fitted to the data from each participant, and the corresponding line is shown in a different color. The blue dashed line is the fit of the simple correlation.

Pearson correlation (*r*, 95% CI; *p* value) was calculated to explore the relationship between variables (Figure 4A). Correlation among devices was statistically significant and very large (*r* [95% CI]: 0.970 [0.970, 0.971], *p* < 0.01; aR^2^ [95% CI]: 0.942 [0.942, 0.943], *p* < 0.01) with strong evidence supporting the alternative hypothesis (BF_10_ > 100). The analysis yielded the following errors: MAE = 1.85 breaths·min^−1^, %Error = 5.24%, and RMSE = 2.42 breaths·min^−1^. In addition, a strong association between measurements was found (r_rm_ [95% CI]: 0.960 [0.959, 0.961]) when analyzing the repeated measures correlation concordance of each soccer player (Figure 4B).

**Figure 5:**
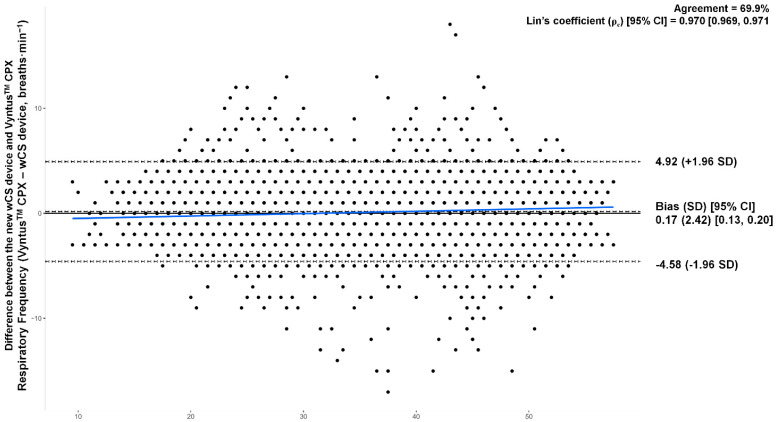
Bland–Altman plot for differences between measured and estimated respiratory frequency (*f*_R_) values. Individual differences between actual and estimated fat mass values are plotted against the mean of measured and estimated fat mass values.

A high concordance was also found (ρ_c_ [95% CI]: 0.970 [0.969, 0.971], bias correction factor: 0.999). Furthermore, a high absolute agreement between methods (ICC [95% CI]: 0.970 [0.970, 0.971]) and a high inter-rater reliability (r_F_: 0.947) were also found. Finally, Vyntus^TM^ CPX, as a standard criterion, showed moderate agreement with the new wCS after Bland–Altman analysis (bias [95% lower to the upper limit of agreement]; % agreement: 0.170 [−4.582 to 4.923]; 69.9%) (Figure 5).

## 4. Discussion

In this study, the aim was to validate a novel wCS for measuring *f*_R_ by comparing the data obtained with a breath-by-breath gas exchange analyzer. Robust time series analysis with Hodges–Lehmann estimation revealed no significant differences between both methods (*p* > 0.05). Moreover, statistical analyses showed a notably strong correlation (aR^2^ = 0.942, *p* < 0.01) and high concordance (ρ_c_ = 0.970, bias correction factor = 0.999) between the new wCS and Vyntus^TM^ CPX, as the criterion method. A bias correction factor is typically used to align estimations more accurately with reference values, aligning the findings more precisely. A factor of one indicates no correction is needed, while factors less than one suggest an underestimation (negative bias), and factors more than one indicate an overestimation (positive bias). The bias correction factor of 0.999 for this new wCS suggests a small adjustment to mitigate a potential bias in the data.

It should be noted that high absolute agreement between methods and high inter-rater reliability was found. Also, the Bland–Altman analysis resulted in a low bias of 0.170 [95% CI from 0.133 to 0.207] breaths·min^−1^. This indicates that a significant proportion of the data points in the analysis fell within an acceptable range of difference between the two measurement methods. Given the device is easy and comfortable to wear, it might become a valuable tool in the real-time evaluation of workload by monitoring *f*_R_ during a soccer training session or match. In fact, in conditions resembling real scenarios, the new wCS showed a smaller overall %Error (5.24%) in the breath-by-breath analysis compared to a recently developed system for the direct measurement of *f*_R_ during exercise (6.65%) [19]. Thus, the new wCS method seems to be valid and reliable for the evaluation of *f*_R_ over a cardiopulmonary exercise test. Considering its low cost, further research might be needed to explore its use in non-professional categories or clinical populations (when physical activity serves as a co-adjuvant for the treatment of certain pathologies).

This study should be analyzed considering the following limitations. First, a greater number of participants might be needed for generalizability; however, it is important to note that participants of this study were elite professional soccer players from Italian Serie A, which implies that the speeds reached during the exhaustion test were high (even over 20 km/h) and the durations were, on average, longer than those of the average population. In line of this, it is advisable to exercise caution when employing the methods interchangeably, especially as additional external validation is still underway. Second, the assessment of *f*_R_ through the novel wCS was carried out without repeated measures, using only one measurement session of the CPET. This naturally reduced the degree to which an evaluation for intra-session reliability of the device could be conducted since the lack of repeated trials may have failed to capture situational factors or individual differences in performance. As well as being of great value, the results of our statistical analyses provided insight into the agreement between the wCS and the reference method. Future studies are recommended to consider multiple trials of other tests within a session as one of the ways to further develop the assessment of reliability and increase the consistency of the measurements across conditions. Thus, the performance of the new wCS must be verified in other field tests. The device seems convenient in terms of wearability without causing disruptions to performance or routine sporting engagements, but this needs to be validated. Our research group already planned a second battery of tests with a protocol that simulates the movements in the field, and this will be a decisive point for the reliability of the instrument; in particular, jumps, changes in direction, or any contact could cause the band to move or alter the signal. It should be underlined, however, that the test was carried out with a completely portable instrument (such as a backpack). Finally, future research is needed in female soccer players to identify a different positioning of the band.

## 5. Conclusions

The novel wCS proves to be a valid and reliable in-field method for assessing *f*_R_ when compared to a breath-by-breath gas exchange analyzer (RMSE = 2.42 breaths·min^−1^). Although the device is easy and comfortable to wear, caution is advised when considering the interchangeability with the criterion method, particularly given the moderate percentage of agreement and the absence of further external validation.

## Figures and Tables

**Table 1:** Device specifications.

Communication Type	Bluetooth (BLE) Long Range *
Allowable distance	130 m (real-time); greater distance under datalogger management (automatic switch)
Features and function mode	Real-time transmission of heart rate (HR) and breath rate (BR), with datalogger function (maximum 1 h) and automatic management/data download upon returning to real-time distance.
Data measurement	Direct measurement of heart rate and respiratory frequency (breath rate) with estimation of VO_2_ and VE
Battery type	Button-style CR 2025
Battery lifetime	Typically 100 h (variable with usage distance)
Operating temperature	−10 °C to +50 °C (14 °F to 122 °F)
Device material	ABS, Nylon PA12, tinned coated brass
Device weight	30 g (with battery installed)
Belt materials	Polyamide and elastane with anti-slip silicone prints

* Supports a maximum of 30 devices simultaneously.

## Data Availability

Data and statistical analyses are available for non-commercial scientific inquiry and/or educational purposes if requested and use does not violate IRB restrictions and/or research agreement terms.
